# Effect of Sugars on *Chlamydia trachomatis* Infectivity

**DOI:** 10.3390/pathogens9040298

**Published:** 2020-04-17

**Authors:** Giacomo Marziali, Antonella Marangoni, Claudio Foschi, Maria Carla Re, Natalia Calonghi

**Affiliations:** 1 FaBiT Department, University of Bologna, Via Irnerio 42, 40126 Bologna, Italy; giacomo.marziali3@unibo.it (G.M.); natalia.calonghi@unibo.it (N.C.); 2 Microbiology, DIMES, University of Bologna, St. Orsola Hospital, Via Massarenti, 9, 40138 Bologna, Italy; antonella.marangoni@unibo.it (A.M.); mariacarla.re@unibo.it (M.C.R.)

**Keywords:** *Chlamydia trachomatis*, sugars, mannitol, sucrose, glucose, elementary bodies, FAK, membranes, LPS

## Abstract

**Background.** Previous works suggest that sugars can have a beneficial effect on *C. trachomatis* (CT) survival and virulence. In this study, we investigated the effect of different sugars on CT infectivity, elucidating some of the molecular mechanisms behind CT-sugar interaction. **Methods.** CT infectivity was investigated on HeLa cells after 2 hour-incubation of elementary bodies (EBs) with glucose, sucrose, or mannitol solutions (0.5, 2.5, 5.0 mM). The effect of sugars on EB membrane fluidity was investigated by fluorescence anisotropy measurement, whereas the changes in lipopolysaccharide (LPS) exposure were examined by cytofluorimetric analysis. By means of a Western blot, we explored the phosphorylation state of Focal Adhesion Kinase (FAK) in HeLa cells infected with EBs pre-incubated with sugars. **Results.** All sugar solutions significantly increased CT infectivity on epithelial cells, acting directly on the EB structure. Sugars induced a significant increase of EB membrane fluidity, leading to changes in LPS membrane exposure. Especially after incubation with sucrose and mannitol, EBs led to a higher FAK phosphorylation, enhancing the activation of anti-apoptotic and proliferative signals in the host cells. **Conclusions.** Sugars can increase CT infectivity and virulence, by modulating the expression/exposure of chlamydial membrane ligands. Further in-depth studies are needed to better understand the molecular mechanisms involved.

## 1. Introduction

*Chlamydia trachomatis* (CT) is the causative agent of the most common bacterial sexually transmitted infection (STI) worldwide [1]. 

CT serovars from D to K are responsible for common uro-genital infections (i.e., cervicitis and urethritis), potentially evolving into serious complications, as pelvic inflammatory disease (PID), and tubal infertility [2].

CT is an obligate intracellular pathogen, characterized by a biphasic development cycle [3]. The extracellular, infectious elementary bodies (EBs) enter epithelial mucosal cells and differentiate into reticulate bodies (RBs) in a membrane bound compartment (i.e. chlamydial inclusion). After several rounds of replication (48–72 h post-infection), intracellular RBs start to re-differentiate into EBs; at the end of the cycle, EBs are released from the host cell, ready to infect neighboring cells [4].

In recent years, several works have focused on the role of sugars during the CT development cycle and its pathogenic process [5,6,7,8]. Omsland and colleagues showed that both EBs and RBs have high levels of metabolic and biosynthetic activity, demonstrating that glucose-6-phosphate is the favorite substrate used by EBs as an energy source [5]. Moreover, it has been shown that glucose-6-phosphate metabolism may be necessary for EB infectious phenotype, as well as for chlamydial lipopolysaccharide (LPS) composition [6,8].

The crucial role of sugars for CT activity has been recently strengthened by a metabolomic analysis performed during the in-vitro interaction between EBs and vaginal lactobacilli: the increased glucose consumption by the most active *Lactobacillus* strains was associated with a significant reduction in EB infectivity [9].

Additionally, it has been found that the concentrations of certain sugars (i.e. sucrose and mannitol) are significantly higher in the urine of women with CT uro-genital infections compared to negative subjects [10]. Thus, higher levels of sucrose and mannitol in the urethral lumen could favor CT acquisition or could be of aid for the bacterial viability [10]. 

In this context, the aim of this study was to assess the effect of different sugars on CT infectivity on epithelial cells. In particular, we analyzed the effect of glucose, sucrose, and mannitol solutions both on EBs and on HeLa cells, with the attempt to elucidate the molecular mechanisms on the basis of CT-sugars interaction.

## 2. Results

### 2.1. Effect of Sugar Solutions on CT Infectivity 

**Figure 1:**
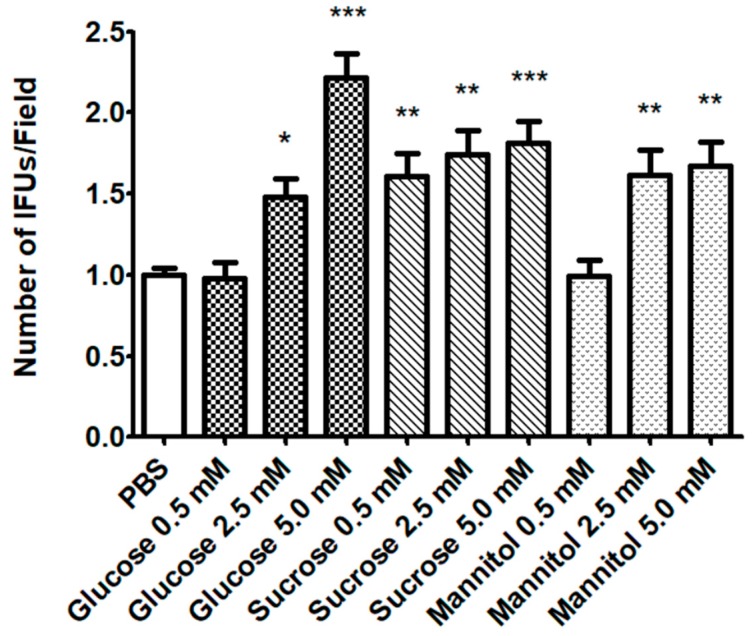
**Evaluation of *C. trachomatis* (CT) infectivity after elementary bodies (EBs) incubation with sugar solutions.** EBs were incubated with different sugar solutions (mannitol, sucrose, glucose; 0.5, 2.5 and 5 mM) for 2 h. Afterwards, HeLa cells were infected at a MOI = 0.1. CT infectivity was evaluated by counting the number of chlamydial IFUs. Results are given as Means ± SD of three independent experiments and are compared to control (PBS; EBs incubated in PBS with no sugars), taken as 1. The asterisks indicate a significant increase in CT infectivity (*, *p* < 0.05; **, *p* < 0.01; ***, *p* < 0.0001) compared to control. Statistical analysis was performed by ANOVA test, followed by Dunnett’s multiple comparison.

The effect of sugar solutions on CT infectivity was verified after EBs-sugar incubation. As shown in Figure 1, all sugars solutions, except for glucose and mannitol 0.5 mM, led to a significant increase of CT infectivity. Glucose solution 5.0 mM showed the highest effect. Globally, considering its significant effect even at the lowest concentration, sucrose exhibited the best activity in enhancing CT infectivity. For all the sugars tested, a dose-response effect was noticed. 

**Figure 2:**
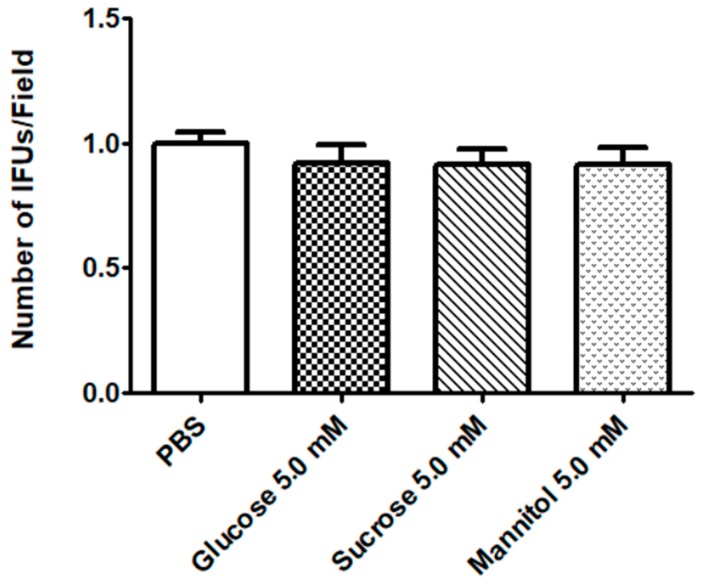
**Evaluation of CT infectivity after HeLa incubation with sugar solutions**. HeLa cells were incubated with different sugar solutions (mannitol, sucrose, and glucose 5 mM) for 2 h and then infected with CT EBs at a MOI = 0.1. CT infectivity was evaluated by counting the number of chlamydial IFUs. Results are given as Means ± SD of three independent experiments and are compared to control (PBS; HeLa incubated in PBS with no sugars), taken as 1. Statistical analysis was performed by ANOVA test, followed by Dunnett’s multiple comparison.

Moreover, we excluded an effect of sugar solutions on HeLa cells: after incubation with glucose, sucrose. or mannitol before CT infection, no effect on EB infectivity was noticed (Figure 2).

### 2.2. Sugar Solutions Increase CT EB Membrane Fluidity 

**Figure 3:**
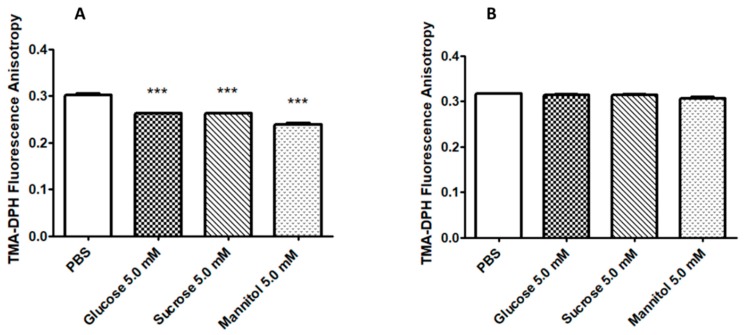
**Evaluation of EBs and HeLa anisotropy after incubation with sugar solutions.** EBs (A) or HeLa cells (B) were incubated with different sugar solutions (mannitol, sucrose, and glucose 5 mM) for 2 h. Afterwards, fluorescence anisotropy was evaluated by the hydrophobic probe TMA-DPH. Results are given as Mean ± SEM of three independent experiments and are compared to control (PBS; EBs or HeLa cells incubated in PBS without sugars). The asterisks indicate a significant modification in fluorescence anisotropy (***, *p* < 0.0001) compared to control. ANOVA test and Dunnett’s multiple comparison were used for the statistical analysis.

After 2 h-incubation with sugar solutions, a significant increase in membrane fluidity (decreased anisotropy values compared to control) was detected for CT EBs (Figure 3A). On the contrary, no effect was noticed on the membrane fluidity of HeLa cells (Figure 3B).

**Figure 4:**
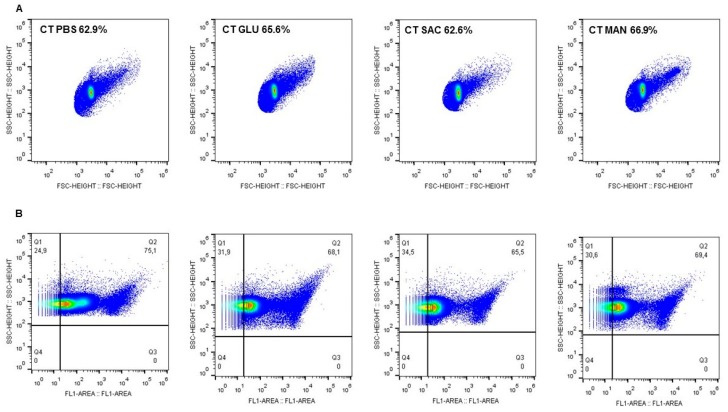
**Quantification of CT EBs by flow cytometry. A**) Dot-plot of the size and granulometry of fluorescent CT EBs after incubation with PBS or glucose (5 mM), sucrose (5 mM), or mannitol (5 mM) for 2 h. **B**) Fluorescence of EBs LPS. A gate was drawn around the LPS-positive cells to calculate the percentages of labelled EBs. Four independent experiments showed similar results.

The increased fluidity induced by sugars was associated with modifications of the EB membrane structure, as demonstrated by flow cytometry experiments with a fluorescent antibody against chlamydial LPS. Figure 4A shows the dot-plot of the size and granulometry of fluorescent CT EBs after incubation with PBS or sugar solutions. Quantification of the labelled cells revealed that 75% of the CT EBs in PBS were LPS-labelled, while the treatment with sugars led to a decrease of LPS-positive EBs (<70%) (Figure 4B). Globally, the reduction of LPS exposure in the case of sugar treatment was statistically significant (*p* < 0.001).

Overall, these data demonstrate that glucose, sucrose, and mannitol solutions increase the membrane fluidity of CT EBs and that this effect leads to a decrease in LPS exposure.

### 2.3. CT EBs Incubated with Sugars Induce FAK Phosphorylation in HeLa Cells. 

We investigated Focal Adhesion Kinase (FAK) phosphorylation state in HeLa cells infected with CT EBs pre-incubated with sugars [11]. Two FAK phosphorylation sites were evaluated: tyrosine 925 (Y925), which is a prerequisite for the anti-apoptotic activity [12], and serine 722 (S722), involved in the transduction of proliferation signals [13].

**Figure 5:**
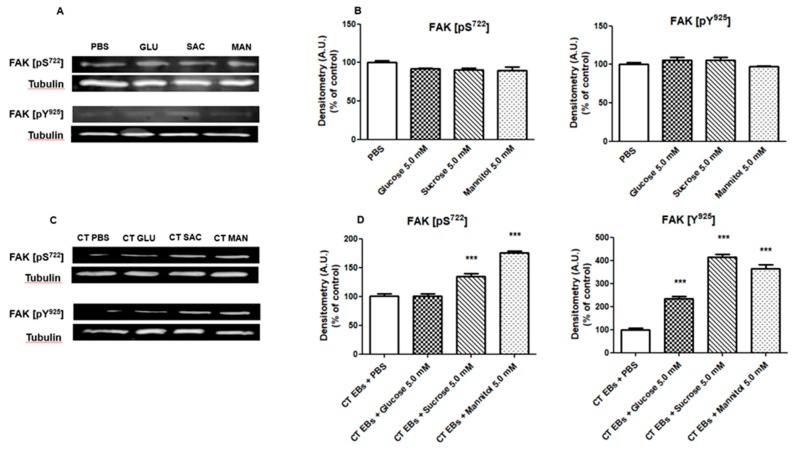
**Phosphorylation state of Focal Adhesion Kinase (FAK) in HeLa cells.** Two FAK phosphorylation sites were explored (i.e. tyrosine 925 -Y925- and serine 722 -S722-), by means of a Western blot analysis. (**A,B**) Hela cells were incubated with PBS or with glucose, sucrose, or mannitol at a concentration of 5 mM for 2 h, and then lysed and analyzed by Western blot. (**C,D**) CT EBs were incubated with PBS or with the different sugar solutions (5 mM) for 2 h and then inoculated in Hela cells for 1 h. Subsequently the cells were lysed and analyzed by Western blot. Relative quantification of FAK pS722/pY925 was performed using the tubulin signal as control. For each condition, densitometry arbitrary units (A.U.) were normalized by CT infectivity values. Results are given as Means ± SD of three independent experiments and are compared to controls (incubation with PBS), taken as 100%. The asterisks indicate a significant increase in FAK phosphorylation (***, *p* < 0.0001) compared to control. Statistical analysis was performed by ANOVA test, followed by Dunnett’s multiple comparison.

As shown in Figure 5C,D, CT EBs incubated with sugar solutions increased significantly the phosphorylation of FAK both on Y925 and on S722 in HeLa cells (*p* < 0.001). The only exception was for glucose 5 mM on S722.

Moreover, we ruled out that sugar solutions per se could change FAK phosphorylation state on HeLa cells: as shown in Figure 5A,B, the incubation of HeLa with PBS or with the different sugars did not change the phosphorylation state of any site.

## 3. Discussion

Previous works suggest that sugars can have a beneficial effect on CT survival, replication, and virulence [7,8,9,10]. However, the exact mechanisms underlying this phenomenon are not yet completely and well understood. Therefore, in this study we investigated the role of different sugar solutions (i.e. glucose, sucrose, and mannitol) on CT infectivity, elucidating some of the physico- chemical and molecular mechanisms behind CT-sugar interaction.

First, we found that sugars can increase CT infectivity, acting directly on chlamydial EBs. In the presence of a dose-response effect, sugar solutions at the highest concentrations tested (5 mM) were able to almost double the number of chlamydial inclusions. In parallel, we ruled out any effect of sugars on the epithelial cells.

These results were subsequently strengthened by the demonstration that sugar solutions significantly increase EB membrane fluidity (reduction of fluorescence anisotropy), with no effect on HeLa cell membranes.

It is known that environmental factors such as pressure, temperature, pH, nutrients, water activity, ions, and enzyme action can change the structure and physico-chemical properties of microbial membranes [14,15,16]. Our results indicate that mannitol, glucose and sucrose can also be significant factors changing the characteristics of CT membranes. 

In this context, it has been found that sucrose and mannitol can increase the stability of chlamydial membranes and proteins (i.e. MOMP: major outer membrane proteins), potentially lengthening the viability of CT EBs [17,18,19]. Higher levels of sucrose and mannitol can be found in urine in case of peculiar dietary habits (i.e. mannitol: consumption of hard candies, fruits and vegetables, sucrose: sugar-rich diet) [20,21]. For that reason, during an uro-genital infection, the presence of these sugars in the urethral lumen could favor CT acquisition or delay its clearance. 

To confirm the hypothesis that sugars can modify the EB membrane, we demonstrated that changes in chlamydial membrane fluidity were associated with a redistribution of EB membrane molecules. In particular, by means of a cytofluorimetric analysis, we found that the presence of sugars led to a significant decrease in LPS exposure.

Chlamydial LPS is involved in bacterial entry into epithelial cells [22,23], and it is essential for secondary differentiation of RBs to infectious EBs [24]. Moreover, it is characterized by a unique lipid A structure, associated with a significantly less stimulatory activity than enteric LPS in inducing proinflammatory signals by human epithelial cells [25,26]. This weak proinflammatory response seems to be related to a poor activation of both the canonical TLR4 and noncanonical cytosolic caspase-11 inflammatory pathways [25]. 

The reduction of LPS expression caused by sugar solutions could further reduce the minimal proinflammatory properties of CT, enhancing the chance of asymptomatic infections in vivo, as a crucial pathogenic strategy.

The reduction in LPS exposure due to a new arrangement of EB membranes could be contemporarily accompanied by a higher expression of chlamydial molecules used as ligands for the entry into the epithelial cells; this could potentially explain the increased CT infectivity induced by sugar solutions. Further in-vitro studies will be crucial to prove that sugars enhance CT infectivity, by modifying the exposure of membrane molecules, used for cellular attachment and internalization (e.g. MOMP, Pmp proteins, Ctad1, OmcB) [3,4]. 

A different sugar-induced expression of EB membrane molecules could also be the basis of the significant higher activation of HeLa signaling pathways found after EB attachment. 

We observed that sugars, especially sucrose and mannitol, increase significantly the phosphorylation of two FAK sites (tyrosine 925 and serine 722) in HeLa cells. At the same time, we excluded that FAK phosphorylation was only due to a higher chlamydial entry into epithelial cells.

FAK is an intracellular protein member of non-receptor tyrosine kinase, activated by an integrin-mediated engagement and involved in cellular adhesion and spreading processes. Its autophosphorylation is a prerequisite to trigger its activity as a signaling protein within cytoskeleton-associated networks. In particular, it has been shown that the phosphorylation of tyrosine 925 is a prerequisite for anti-apoptotic activity [12], whereas the phosphorylation of serine 722 is involved in the transduction of proliferation signals [13].

Considering that the remodeling of the host cell actin cytoskeleton is usually required for efficient bacterial invasion, there has been increasing interest in the role of FAK in the link between microbial recognition and the initiation of pro-inflammatory responses. Previous works showed the role of FAK in the invasion and internalization of different microorganisms, including *Yersinia enterocolitica*, *Listeria monocytogenes*, *Campylobacter jejuni*, *Neisseria meningitidis,* and *Salmonella typhimurium* [11,27,28,29].

FAK was also reported to be involved in invasion-mediated uptake of chlamydiae; it has been shown that integrins, key receptors of the chlamydial pathogenic process, come directly in contact with the pathogen through their extracellular domains and then bind their intracellular β-tails to FAK [30,31,32]. 

Globally, we can speculate that sugars induce a higher exposure of EB ligands able to activate FAK pathways in HeLa cells. In this way, chlamydial EBs can enhance the activation of anti-apoptotic and proliferative signals in the host cells, favoring their infectivity and survival into the host cells. 

Further studies including a larger panel of CT serovars are needed to understand if our observations can be extended to all chlamydial serovars. We can speculate that the effect of sugars is not limited to one specific serovar: indeed, in a previous work [10], we found that women with CT uro-genital infections are characterized by higher levels of urinary sucrose, and mannitol, irrespective of the chlamydial serovars.

Moreover, new in-depth experiments will be necessary to shed light on the exact changes taking place on EB membrane molecules after sugar interaction. 

## 4. Materials and Methods

### 4.1. Chlamydia trachomatis Strain and Cell Culture 

CT strain GO/86 (serovar D) was used for the experiments. This clinical strain was isolated from a patient with urethritis and belongs to the laboratory collection of the Microbiology Unit of S. Orsola-Malpighi Hospital of Bologna (Italy). The strain was initially propagated for about 2  weeks in LLC-MK2 cells (ATCC^®^ CCL-7TM). Afterwards, CT EBs were purified by Renografin density gradient centrifugation [32,33]. The infectivity titer (expressed as inclusion forming units-IFU/mL) was determined in HeLa cells, as described elsewhere [33,34]. 

Molecular genotyping of the strain was performed by *omp1* gene sequencing [35].

HeLa cells (ATCC® CCL-2), originated from a human cervix adenocarcinoma, were used for the experiments. Cells were grown in 6 well plates containing sterile coverslips (Thermo Fisher Scientific, Waltham, MA), in DMEM medium (EuroClone, Pero, Italy; added in with 10% fetal bovine serum and 1% L-glutamine, with no antibiotics–‘complete medium’), in 5% CO_2_ at 37 °C. 

### 4.2. Evaluation of CT Infectivity after EB Incubation with Sugar Solutions 

To study the ability of sugar solutions to directly enhance CT EB infectivity, infection experiments were performed as follows. 

Sugar solutions of glucose, sucrose, and mannitol were prepared from powder stocks (Sigma Aldrich) in sterile phosphate buffer saline (PBS) and then diluted to final concentrations of 5 mM, 2.5 mM, and 0.5 mM. HeLa cells were seeded in 6-wells plates in 2 mL of complete medium and allowed to reach a total cell number of 5 × 10^5^ approximately. 

A total of 5 × 10^4^ CT EBs (10 µL of a stock solution of 5 × 10^3^ EBs/µL) were re-suspended in 1 mL of sugar solutions and then incubated for 2 h at 37 °C with 5% CO_2_. A PBS sterile solution was used as negative control. After the incubation, the complete medium was removed and EB solutions were used to infect HeLa cells for 1 h [multiplicity of infection (MOI) = 0.1]. No centrifugation steps were included, as well as no cycloheximide being added to the culture medium in order to better mimic a natural infection. At the end of the incubation, each plate was PBS-washed three times, and 2 mL of complete medium were added. Plates were then incubated at 37 °C with 5% CO_2_ for 48 h. All the experiments were conducted in triplicate.

CT infection was estimated by counting the number of IFUs by direct immunofluorescence, using a fluorescein-conjugated anti-chlamydial LPS monoclonal antibody (Meridian, Cincinnati, OH, United States) [33]. The number of IFUs was counted in 60 randomly chosen 40× microscopic fields.

### 4.3. Evaluation of CT Infectivity after HeLa incubation with Sugar Solutions

To exclude an effect of sugar solutions on HeLa cells, infection experiments were performed as follows. HeLa cells (5 × 10^5^ cells approximately) were incubated with sugar solutions (only 5 mM) for 2 h, at 37 °C, with 5% CO_2_. After the incubation, the cell plates were washed twice with PBS, and infected with CT EBs (MOI = 0.1) for 1h. Next experimental steps were conducted as described before.

### 4.4. Fluorescence Anisotropy Measurements

Steady-state fluorescence anisotropy was used to investigate the possible modifications induced by PBS or sugar solutions on the physico-chemical state of CT EB membranes. The membrane fluidity of HeLa cells and CT EBs was estimated by measuring fluorescence anisotropy of the hydrophobic probe TMA-DPH [1-(4-trimethylammoniumphenyl)-6-phenyl-1,3,5-hexatriene p-toluenesulfonate] (ThermoFisher Scientific, Waltham, MA). TMA-DPH is a lipophilic fluorophore that penetrates the membrane hydrophobic core, orienting perpendicularly to the membrane plane [36]. In case of an increased fluidity of the membrane, the TMA-DPH probe rotates to a greater extent, leading to a depolarization of the fluorescence emission and a decrease in the fluorescence anisotropy. 

Anisotropy was measured after 2 h-incubation with sugar solutions (glucose, sucrose, and mannitol 5 mM) for 2 h, at 37 °C, with 5% CO_2_. A PBS solution without sugars was used as a control. 

HeLa cells and EBs were suspended at a final concentration of 3 × 10^5^ cells/mL and 5 × 10^4^ cells/mL, respectively. For the anisotropy measurements, the samples were incubated with TMA-DPH and then analyzed by a PTI QuantaMaster fluorimeter (Photon Technology International, North Edison, NJ, USA) according to Parolin et al. [30].

### 4.5. Flow Cytometry 

To verify if the increased fluidity induced by sugars could modify the membrane structure, CT EBs were incubated with PBS or sugar solution, stained with an anti-LPS antibody, and analyzed by flow cytometry. 

A total of 2 × 10^5^ CT EBs was re-suspended in 1 mL of sugar solutions (only 5 mM) and incubated for 2 h at 37 °C with 5% CO_2_. EBs diluted in sterile PBS were used as a negative control. After the incubation, EBs were stained in solution using a fluorescein-conjugated anti-chlamydial LPS monoclonal antibody (Meridian, Cincinnati, OH, United States). CT EBs were then centrifuged for 1 h at 40,000 × g and re-suspended in sterile PBS. Samples were then analyzed by S3e Cell Sorter (Bio-Rad, Hercules, CA, United States), following the manufacturer’s instructions. As a negative control, CT EBs were incubated with FITC-mouse anti-human IgG.

### 4.6. Cell Lysis and Western Blot Analysis 

To evaluate modifications of intracellular signaling pathways during the early phase of CT infection, we analyzed the phosphorylation state of two sites of FAK (i.e. tyrosine 925 (Y925) and serine 722 (S722)), a non-receptor tyrosine kinase involved in invasion mediated bacterial uptake and subsequent pro-inflammatory responses [11,12,13].

Cell lysis and Western blot experiments were performed as follows. HeLa cells were seeded in 6-wells plates in 2 mL of complete medium and allowed to reach a total cell number of 5 × 10^5^ approximately. 

A total of 5 × 10^4^ CT EBs (10 µL of a stock solution of 5 × 10^3^ EBs/µL) were re-suspended in 1 mL of sugar solution (only 5 mM), and then incubated for 2 h, at 37 °C, with 5% CO_2_. EBs diluted in PBS sterile solution, as well as sugar solutions without EBs were used as controls. After the incubation, the complete medium was removed and EB solutions were used to infect HeLa cells for 1 h (MOI = 0.1). 

Cells were washed with ice-cold PBS and lysed according to Parolin et al. [30]. In brief, lysed cells were centrifuged at 12,000 × g for 20 min at 4 °C, and protein concentration was determined by using the Bio-Rad protein assay (Bio-Rad, Hercules, CA, United States). The proteins were resolved by SDS-PAGE and immunoblotted with a rabbit anti-human FAK pS722/pY925 (1:1000 in PBS), or with a mouse anti-tubulin (1:5000 in PBS) antibodies.

Detection of immunoreactive bands was performed with a secondary antibody (1:10,000 in PBS Tween) conjugated with horseradish peroxidase (GE Healthcare, Milan, Italy), and developed with WESTAR EtaC 2.0 (Cyanagen, Bologna, Italy). Densitometry analysis was performed by Fluor-S Max MultiImager (Bio-Rad, Hercules, CA, United States). Relative quantification of FAK pS722/pY925 was done by using tubulin signal as a control. For each condition, densitometry arbitrary units (A.U.) were normalized by CT infectivity values.

### 4.7. Statistical Analysis 

Statistical analyses were conducted using GraphPad Prism software (GraphPad Prism version 5.02 for Windows, GraphPad Software, San Diego California USA, www.graphpad.com). Results are expressed as Means ± Standard Deviation (± SD) of a series of independent experiments. Data were analyzed by ANOVA test, followed by post-hoc Multiple Comparison tests (i.e. Dunnett’s test). *p* < 0.05 (*), *p* < 0.01 (**) and *p* < 0.0001 (***) were considered statistically significant.

## 5. Conclusions

We found that sugars (i.e. glucose, sucrose, and mannitol) are able to increase CT infectivity on epithelial cells, acting directly on the EB structure. Sugars induce a significant increase of EB membrane fluidity, leading to changes in LPS expression on chlamydial membranes.

After incubation with sugar solutions, chlamydial EBs lead to a higher phosphorylation of FAK, enhancing the activation of anti-apoptotic and proliferative signals in the host cells.

Further in-depth analyses are needed to shed light on the molecular mechanisms involved in the interaction between CT, sugars, and the host cells.
